# Diabetic Foot Ulcer Prevention Priorities Established by Consumers From Low Socioeconomic and Culturally Diverse Communities

**DOI:** 10.1002/dmrr.70206

**Published:** 2026-08-04

**Authors:** Jayishni N. Maharaj, Jim Woodburn, Annette McLaren‐Kennedy, Mohamed A. Kola, Daniel M. G. Winson, Meegan Nevin, Kelly Clanchy

**Affiliations:** ^1^ Australian Centre for Precision Health and Technology (PRECISE) Griffith University Gold Coast Queensland Australia; ^2^ School of Allied Health Sport and Social Work Griffith University Gold Coast Queensland Australia; ^3^ Logan Endocrine and Diabetes Services (LEADS) Logan Hospital Metro South Health Meadowbrook Queensland Australia; ^4^ The Hopkins Centre Griffith University Nathan Queensland Australia

**Keywords:** co‐design, consumer, diabetes, equity, prevention, ulcer

## Abstract

**Aims:**

Diabetic foot ulcers (DFU) continue to occur despite established prevention guidelines, yet consumer perspectives, particularly from socioeconomically disadvantaged and culturally and linguistically diverse (CALD) communities, have been underrepresented in shaping prevention. To address this gap, we aimed to co‐design a consumer‐derived prevention research agenda by identifying barriers and enablers, establishing research priorities, prioritising implementation strategies, and defining meaningful outcome measures.

**Methods:**

Four sequential workshops were conducted with 11 consumers with a history of DFU in South East Queensland, including Aboriginal and Pacific Islander participants (*n* = 4) and those from areas of socioeconomic disadvantage (*n* = 8). Rapid qualitative analysis informed iterative refinement across workshops, with priorities, implementation strategies, and outcome measures ranked by participants.

**Results:**

Participants identified barriers across clinical, self‐care, psychosocial, social, and financial domains, including feeling unsupported when trying to prevent DFU, the cognitive burden of constant vigilance, conflicting guidance, and the financial and travel costs of care. The highest‐ranked research priorities were better ways to predict problems before they happen (median 2, IQR 1–4.5), formal education support (median 4, IQR 3–9), and tools to support regular foot checks (median 5, IQR 3–6). Across all three priorities, consumers consistently selected technology enabled implementation strategies, including wearable sensors, AI powered personalised learning, and smart interventions. Participants defined prevention success through quality of life, function, and confidence outcome measures rather than clinical endpoints alone.

**Conclusions:**

For socioeconomically disadvantaged and CALD populations, effective DFU prevention must be technology enabled, personalised, and developed in partnership with the consumer.

## 1 Introduction

Diabetic foot ulcers (DFUs) are a major driver of morbidity, loss of mobility, and reduced quality of life, and remain one of the most serious complications of diabetes. Over the past decade, prevention guidance has become increasingly clear, emphasising risk identification, regular surveillance, protective footwear and offloading, and timely access to specialist care [1]. However, ulceration rates have remained largely unchanged or increased in roughly half of the countries studied over the past 2 decades [2], and recurrence rates remain high at approximately 42% within 1 year and 65% within 5 years of healing [3, 4]. This indicates a persistent gap between the goals of prevention guidance and what is achieved in practice.

DFU prevention has largely been shaped by clinicians, researchers, and policy bodies [5]. Recent priority‐setting work has begun to broaden whose voices contribute to the prevention agenda, including a national Delphi study of Australian stakeholders that identified markedly different research priorities between consumers and health professionals, with no shared questions in their respective top 10 lists [5, 6]. However, consumers from culturally and linguistically diverse (CALD) communities and socioeconomically disadvantaged populations remain particularly underrepresented in shaping prevention [5, 6] while carrying a disproportionate burden of disease. Type 2 diabetes is around three to four times more prevalent among Aboriginal and Torres Strait Islander peoples [7] and, after adjustment for age and socioeconomic status, 6.3 to 7.2 times higher in Pacific Island–born Australians [8]. Diabetic foot ulceration and amputation are three‐ to six‐fold more common in Aboriginal and Torres Strait Islander Australians [7], and are also more common among people on lower incomes and in the most disadvantaged areas [9]. Underrepresentation matters because consumers carry the daily work of prevention, and that work looks different across populations. What is feasible, meaningful, or sustainable for one group may not hold for another [10]. Defining DFU prevention without their perspectives risks producing strategies, guidance, and outcome measures that do not reflect what works for the people who need prevention most.

To address this gap, we used a co‐design approach with consumers at high risk of DFU, including Aboriginal and Pacific Islander participants and those from areas of socioeconomic disadvantage. This study aimed to (i) characterise consumer‐perceived barriers and enablers to DFU prevention, (ii) establish consumer‐derived prevention research priorities, (iii) identify and prioritise implementation strategies for the highest‐ranked priorities, and (iv) prioritise outcome measures for DFU prevention research.

## 2 Materials and Methods

### 2.1 Study Design

This study used a qualitative descriptive design embedding the principles of co‐design to inform research and clinical priorities for DFU prevention. Ethics approval was granted by the Metro South Health Human Research Ethics Committee (HREC/2025/QMS/117922) and the Griffith University Human Research Ethics Committee (2025/797) and the study was conducted in accordance with the Declaration of Helsinki.

### 2.2 Participants

Eleven consumers with a history of DFUs participated in the workshops (8 male; 3 female; 2 type 1 diabetes; 9 type 2 diabetes; 28 to 74 years of age). Eleven participants attended Workshop 1. One participant withdrew after Workshop 1, and the remaining 10 participants attended Workshops 2, 3, and 4. The reason for participant withdrawal was not communicated with the research team. Participants identified as Australian (*n* = 6, 55%) or as Aboriginal and/or Pacific Islander (*n* = 4, 36%). Postcode data indicated that 80% of participants (*n* = 8) resided in areas of relative socioeconomic disadvantage (IRSD deciles 1 to 3) based on SEIFA 2021 classifications [11]. One participant did not provide their postcode and cultural background. Participants were recruited by a researcher (MAK) who was not involved in workshop facilitation to minimise potential bias.

### 2.3 Data Collection

Data were collected through four face‐to‐face workshops conducted in 2025 at a High Risk Foot Clinic. Each session lasted approximately two hours and was facilitated by author (KC), an experienced qualitative researcher. The workshops were conducted sequentially, with findings from each workshop informing subsequent data collection. The sequential structure supported exploration of experiences, prioritisation of prevention strategies, and refinement of findings. All sessions were audio‐recorded.

**FIGURE 1:**
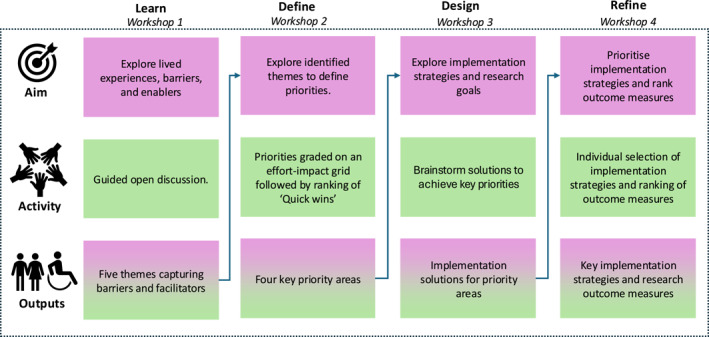
Schematic illustrating a sequential co‐design process spanning four workshops aligned with the phases Learn, Define, Design, and Refine. Workshops progressed from exploring lived experiences, including barriers, and enablers, to prioritising action areas, generating implementation solutions, and ranking research outcome measures. For each workshop, aims (top box), activities (middle boxes), and outputs (bottom boxes) are shown.

The overall co‐design process and workshop outputs are summarised in Figure 1. Workshop 1 explored consumer experiences of DFU prevention and identified perceived enablers and barriers. The Theoretical Domains Framework (TDF) was used to develop the questions that guided Workshop 1 facilitation [12]. Workshop 2 reviewed preliminary themes identified during Workshop 1 to define DFU research priorities. Thirty priority research areas were compiled from three sources: (1) 15 priorities derived from participant experiences in Workshop 1, (2) 10 priorities previously identified by JNM and JW based on their clinical and research expertise in DFU prevention, and (3) 5 derived from Goals 1 to 5 of the 2030 Australian Strategy for Foot Health and Disease in Diabetes [13]. During Workshop 2, the facilitator (KC) led a group discussion on each priority, and participants collaboratively determined its placement on a difficulty‐impact matrix through real time group consensus. Participants then ranked the priorities positioned in the high impact, low effort quadrant in order of importance as an individual paper based task. Workshop 3 focused on brainstorming and refining implementation strategies to address the key priority areas. Strategies were documented in real time and carried forward to Workshop 4. In Workshop 4, participants selected a preferred implementation strategy for each of the key priority areas. Participants were subsequently provided with a list of possible research outcomes for DFU research and asked to rank these using an individual paper‐based task. Rankings were collated to identify the highest‐priority strategies and outcome measures.

After the workshops, participants completed an evaluation comprising 17 Likert items assessing the extent to which they felt listened to, valued, and comfortable, the perceived importance of their contribution, and the structure of the sessions, plus two open‐ended questions on workshop strengths and areas for improvement.

### 2.4 Analysis

Analysis was conducted concurrently with data collection to support the iterative workshop design. A rapid qualitative analysis approach was used to allow emerging findings to inform subsequent workshops. Rapid data analysis is a streamlined process for qualitative data collection and analysis, focussing on efficient analysis of data to derive actionable insights quickly [14, 15]. This approach was selected based on the requirement to quickly apply workshop findings to inform subsequent workshops.

Analysis of Workshop 1 focused on identifying perceived enablers and barriers to DFU prevention. Audio recordings were transcribed verbatim using an AI based transcription service. Transcripts were manually checked for accuracy and anonymised by AMK. Field notes recorded independently by AMK and JNM were used to support contextual interpretation. Transcript excerpts were initially coded to TDF domains by AMK to systematically organise the data. However, during collaborative analysis, the research team identified that the TDF domains did not adequately capture the distinct barriers and enablers described by participants. Themes were therefore developed inductively across the coded data, resulting in five domains that the research team considered more representative of participants' prevention experiences [16]. Related participant statements from workshop transcripts and workshop discussions were grouped, and recurrent patterns influencing experiences of DFU prevention were defined as themes representing barriers and enablers. Following Workshop 2, transcripts, field notes, and workshop outputs were reviewed to refine preliminary themes and to further identify key barriers and enablers.

Quantitative analysis was conducted for Workshops 2, 3 and 4 to summarise prioritisation outputs. For each priority and outcome measure, median scores and interquartile ranges were calculated to summarise prioritisation and assess agreement across participants. Interquartile ranges are reported as the 25th to 75th percentiles. For Workshop 3, participants voted for the implementation strategies they considered would have the greatest impact on each of the top three priorities; some participants selected more than one strategy per priority. Votes were summarised as counts and as a percentage of total votes cast for each priority.

Responses to the post‐workshop evaluation Likert items were expressed as a percentage of total responses. Responses to the open‐ended questions were analysed using content analysis to identify key themes and patterns.

### 2.5 Rigour

Analytic rigour was supported through researcher and data triangulation. KC facilitated the workshops, while AMK and JM independently recorded field notes and contributed to analysis. Findings were reviewed iteratively across workshops using transcripts, field notes, and structured workshop outputs. None of the workshop facilitators were involved in the clinical care of participants, reducing potential power imbalances. AMK's personal connection to diabetes, without professional or lived experience of DFUs, was acknowledged and considered during analysis.

### 2.6 Use of Artificial Intelligence

Generative AI (Claude, Anthropic, San Francisco, CA, USA) was used to assist with copyediting and refinement of prose during manuscript preparation. All content, analyses, interpretations, and conclusions are the work of the authors.

## 3 Results

### 3.1 Barriers and Enablers to DFU Prevention

Participants identified barriers and enablers to DFU prevention across five domains, including: clinical, self‐care, psychosocial, social, and financial (Table 1). Clinical enablers included self‐advocacy and active engagement in care. Clinical barriers related to fragmented and lack of whole‐person care, limited specialist availability, delayed access, feeling unsupported and conflicting or unclear guidance. Self‐care enablers included routine foot checks, use of appropriate footwear and moisturisers, self‐directed strategies including daily routines, experimentation with offloading strategies, and self‐directed learning from peers and online resources. Cognitive burden and prior experience influenced engagement in preventive self‐management and decision‐making behaviours. Psychosocial barriers were prominent and included cognitive and emotional burden, anticipatory distress, difficulties with understanding and correctly applying information, limiting the scope and success of efforts, and reduced confidence in prevention. Participants identified a sense of personal responsibility, self‐blame, and practitioner blame for DFU occurrence, with fear of ulcer recurrence, amputation, or loss of mobility identified as a motivator for some participants. Social support enabled preventive care, while loss of independence, sense of self and role and social isolation acted as barriers. Financial barriers including costs of care and activities of daily living, medical appointment and travel burden and difficulties maintaining employment were identified as negatively influencing DFU prevention. Participants identified that proximity to healthcare services positively influenced DFU prevention efforts.

**TABLE 1:** Enablers and barriers to diabetic foot ulcer prevention.

Clinical	Barriers Limited integration of whole‐of‐person considerations in clinical care. Competing priorities between limb preservation and overall survival. Limited access to experienced foot‐care clinicians. Delayed access to timely and appropriate care. Feeling unsupported or “flying blind” for preventing complications. Misleading information regarding management of diabetes. Conflicting exercise recommendations during ulcer management. Lack of individualised dietary and exercise guidance. Not knowing why ulcers recur influencing confidence in self‐management.
Self‐care	Enablers Regular foot checks supporting early identification of problems. Use of moisturisers, appropriate footwear, and daily routines. Experimentation with offloading strategies. Self‐directed learning and knowledge‐seeking through peers and online resources. Barriers Cognitive load associated with constant self‐monitoring and decision‐making. Prior experience with DFU, determines self‐management complexity and level of active engagement in management strategies.
Psychosocial	Enablers Fear of ulcer recurrence, amputation, or loss of mobility motivated engagement in preventive behaviours. Barriers Cognitive and emotional burden of constant vigilance, eventually becoming hypervigilance. Emotional burden of prolonged immobility and treatment restrictions. Cycle of fear, grief, and anticipatory distress. Compounding emotional burden of recurrent ulceration. Medical jargon and unclear explanations limiting effective self‐care. Misattribution of causes (e.g. focus on sugar alone) complicating prevention efforts. Overwhelming sense of personal responsibility and emotional exhaustion. ◦ Self‐blame when ulcers recur despite best efforts. ◦ Feeling blamed by health professionals despite best efforts.
Social	Enablers Social support enabling routine preventive foot care. Barriers Loss of independence and sense of self and role. Isolation from wider social networks.
Financial	Enablers Proximity to healthcare services. Barriers Financial cost of preventative foot care. Increased costs associated with activities of daily living. Burden of medical appointments. Travel‐related costs associated with accessing healthcare. Difficulty maintaining employment due to treatment demands and mobility restrictions.

### 3.2 Priority Research Areas for DFU Prevention

**FIGURE 2:**
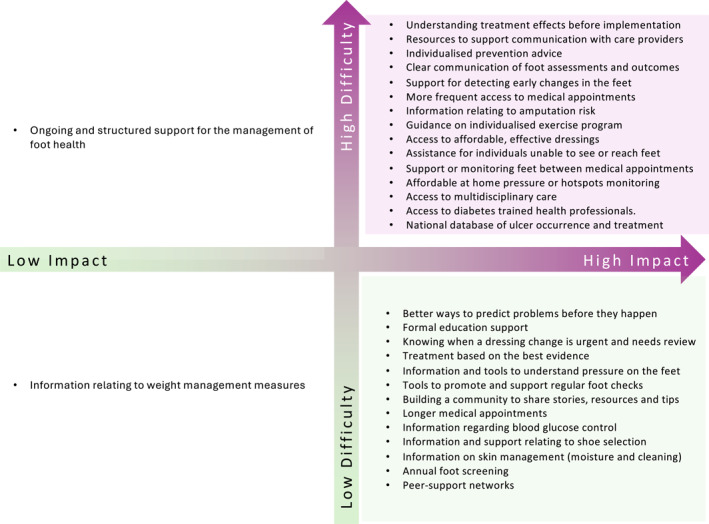
Difficulty–impact matrix used to map priorities for diabetic foot ulcer prevention. Priorities identified from Workshop 1 were positioned according to perceived impact (horizontal axis; low to high) and effort required to address them (vertical axis; low to high). Priorities in the high‐impact, high‐effort quadrant represent major areas requiring substantial investment, while those in the high‐impact, low‐effort quadrant indicate quick or easy wins.

Thirty priority research areas for DFU prevention were identified by researchers and participants. Participants collaboratively mapped the priority research areas onto an effort–impact matrix (Figure 2). Of the 30 priorities identified, 93% were positioned in the high‐impact quadrants. Half of all priorities (50%) were classified as high impact but high effort, while 43% were classified as high impact and low effort. Participants were asked to rank priorities located in the high‐impact, low‐effort quadrant in order of importance to identify and prioritise immediate research opportunities (Table 2). The highest‐ranked priority, better ways to predict problems before they happen, had the lowest median score and a narrow interquartile range (median 2, IQR 1 to 4.5), indicating strong agreement among participants. Formal education support ranked second (median 4, IQR 3 to 9). Tools to promote and support regular foot checks (median 5, IQR 3 to 6) and accessible education relating to foot ulcer prevention (median 5, IQR 4 to 7.5) returned equal median scores. Four priorities, rather than three, therefore progressed as top ranked.

**TABLE 2:** High‐impact, low‐effort priorities for diabetic foot ulcer prevention.

Priorities	Participants										Median (IQR)
	1	2	3	4	5	6	7	8	9	10	
Better ways to predict problems before they happen	2	6	1	1	1	8	1	2	3	5	2 (1–4.5)
Formal education support	4	3	2	9	9	3	13	11	4	1	4 (3–9)
Tools to promote and support regular foot checks	3	11	6	5	6	5	3	7	2	2	5 (3–6)
Accessible education relating to foot ulcer prevention	5	4	5	8	10	13	4	4	6	4	5 (4–7.5)
Knowing when a dressing change is urgent	11	7	7	4	2	1	5	3	11	6	5.5 (3.25–7)
Annual foot screening	1	9	4	3	8	6	11	6	1	13	6 (3.25–8.75)
Treatment based on the best evidence	9	2	11	10	3	2	7	8	7	7	7 (4–8.75)
Information and tools to understand pressure on the feet	7	8	9	6	7	7	10	9	10	3	7.5 (7–9)
Information regarding blood glucose control	6	5	3	11	11	11	8	1	12	8	8 (5.25–11)
Information relating to skin management	8	10	8	12	5	12	6	10	13	9	9.5 (8–11.5)
Longer medical appointments	10	13	10	2	13	10	12	5	8	10	10 (8.5–11.5)
Building a community to share stories, resources and tips	12	12	13	7	4	9	9	13	9	11	10 (9–12)
Peer‐support networks	13	1	12	13	12	4	2	12	5	12	12 (4.25–12)

*Note:* Priorities are located in the high‐impact, low‐effort quadrant of the effort–impact matrix (Figure 2). Participants ranked these priorities from 1 (highest) to 13 (lowest). Median ranks and interquartile ranges were calculated to summarise prioritisation and assess agreement across participants, with lower median values indicating higher priority. Cell shading reflects the strength of each participant's ranking, with darker green indicating priorities ranked closer to 1 (highest priority) and lighter or no shading indicating lower‐ranked priorities.

Goals 1 to 5 of the 2030 Australian Strategy for Foot Health and Disease in Diabetes were embedded alongside participant‐derived priorities. Three national goals, access to preventative footcare (Goal 2), access to interdisciplinary high risk foot services (Goal 3), and national outcome reporting (Goal 5), were placed in the high impact, high difficulty quadrant. The remaining two were prioritised as low effort and high impact and therefore ranked in order of priority by consumers: annual foot screening (Goal 1; ranked 6th of 13; median 6, IQR 3.25 to 8.75) and treatment based on the best evidence (Goal 4; ranked 7th of 13; median 7, IQR 4 to 8.75).

### 3.3 Priority Implementation Strategies

Implementation strategies were identified by researchers and participants and prioritised by participants for the top three DFU prevention research priorities. Participants were asked to vote for the implementation strategy they thought would have the greatest impact on the identified priority. Better ways to predict problems before they happen, education workshops on spotting small foot changes and wearable neural sensors that replicate nerve function were equally ranked first (3 votes each; 30% of votes), followed by smart socks or shoes with continuous monitoring and AI‐enabled predictive models (2 votes each; 20%). Formal education support, an education program commencing at diagnosis was ranked first (6 votes, 43%), followed by AI‐powered personalised micro‐learning (5 votes, 36%) and digital twin simulation of a patient's foot forecasting outcomes based on behaviour (3 votes, 21%). Tools to promote and support regular foot checks, smart flooring mats detecting plantar hot spots were ranked first (7 votes, 54%), with AI visual analysis of daily foot photos and smart mirrors highlighting abnormalities equally ranked second (3 votes each, 23%).

### 3.4 Priority Outcome Measures

Participants prioritised outcome measures for DFU prevention research based on perceived importance for evaluating prevention success (Table 3). Outcome measures prioritised most highly were patient‐centred and functional in nature. Improvements in daily quality of life had the lowest median score (median 3, IQR 1–6.5), indicating the highest prioritisation among participants. Outcomes related to functional ability and confidence were also highly prioritised, including the ability to walk safely and confidently (median 4.5, IQR 3–7.5) and feeling confident in preventing future ulcers (median 4.5, IQR 2.25–8.75). In contrast, outcomes focused on clinical metrics and treatment processes were prioritised lower. These included staying ulcer‐free at 12 months (median 9, IQR 6.25–14.5), pressure reduction on the healed ulcer (median 11, IQR 9–13), and how often prescribed footwear is worn (median 14, IQR 9.25–15.75).

**TABLE 3:** Outcome measures for diabetic foot ulcer prevention research.

Outcome measures	Participants										Median (IQR)
	1	2	3	4	5	6	7	8	9	10	
Improvements in daily quality of life	1	1	14	5	7	3	15	1	1	3	3 (1–6.5)
Ability to walk safely and confidently	4	5	3	14	9	2	2	8	3	6	4.5 (3–7.5)
Feeling confident in preventing future ulcers	3	9	2	8	16	5	16	2	4	1	4.5 (2.25–8.75)
Any new foot problems developing	2	4	1	7	11	1	12	12	12	2	5.5 (2–11.75)
Pain levels in the feet	5	16	10	4	13	6	5	4	2	7	5.5 (4.25–9.25)
How easy it is to check your feet at home	8	2	4	12	12	14	1	3	15	5	6.5 (3.25–12)
New areas of pressure or callus forming	7	3	7	6	10	7	14	11	16	4	7 (6.25–10.75)
How healthy the healed area looks on the inside	6	10	8	1	2	11	9	15	5	8	8 (5.25–9.75)
Staying ulcer‐free at 12 months	16	15	13	10	4	8	6	5	7	15	9 (6.25–14.5)
Staying ulcer‐free at 12 weeks	11	13	11	2	1	9	7	7	9	13	9 (7–11)
Staying ulcer‐free at 6 months	15	14	12	9	3	10	8	6	8	14	9.5 (8–13.5)
Daily easy of offloading treatment	14	8	9	3	8	16	3	13	14	12	10.5 (8–13.75)
Comfort of the prescribed shoes or padding	12	6	15	11	6	4	10	10	13	11	10.5 (7–11.75)
Pressure reduction on the healed ulcer	9	11	6	15	NA	12	13	16	6	9	11 (9–13)
How much the pressure improves compared to before	10	12	5	13	5	13	11	14	11	10	11 (10–12.75)
How often the prescribed footwear is worn	13	7	16	16	15	15	4	9	10	16	14 (9.25–15.75)

*Note:* Participants ranked all outcome measures from 1 (highest) to 16 (lowest). Median ranks and interquartile ranges were calculated to summarise prioritisation and assess agreement across participants, with lower median values indicating higher priority. NA indicates not voted upon. Cell shading reflects the strength of each participant's ranking, with darker green indicating outcome measures ranked closer to 1 (highest priority) and lighter or no shading indicating lower‐ranked measures.

### 3.5 Participant Experience

Six participants provided qualitative feedback regarding the strengths of the workshop and areas requiring modification. Three participants identified communication as a strength of the workshops, specifically the friendly and open nature of their peers and the workshop facilitators. The knowledge and facilitation skills of the facilitator were identified as a strength by two participants. Two participants identified exposure to peer knowledge, specifically sharing stories, strategies and skills relating to foot care as a strength, with one participant identifying that participating in the workshop improved their life as a person living with diabetes foot disease. No participant identified areas that required modification or change to improve their experience. Overall participants felt listened to, valued and comfortable; felt their ideas were given serious consideration and that the goals, structure and outcomes of the workshop were clear.

## 4 Discussion

Drawing on the lived experience of consumers at high risk of DFU, including Aboriginal and Pacific Islander participants and those from areas of socioeconomic disadvantage, this co‐design study demonstrates that effective DFU prevention must be technology enabled, personalised, and developed in partnership with the consumer. Across all four priority research directions (better prediction of problems, formal education support, tools for regular foot checks and accessible education), consumers consistently selected strategies with a focus on technology‐based solutions. These strategies were prioritised as a mechanism for reducing the daily burden of monitoring, decision making, and learning that prevention currently demands. Consumers also made clear that these strategies will only work if personalised to their medical history, life circumstances, and competing demands. Together, these findings point to technology enabled and personalised strategies developed in partnership with consumers as the priority for the next generation of DFU prevention.

Participants identified a lack of individualised clinical guidance as a barrier for DFU prevention, with current advice often conflicting or disconnected from their broader circumstances. Participant priorities and definitions of prevention success focused on the personal level, what makes prevention work in daily life, including maintaining function, confidence, and quality of life. Participants also noted that prior experience with DFU shaped the complexity of self‐management, meaning that prevention support cannot assume a uniform starting point. Van Netten and colleagues [17] have called for a shift towards personalised medicine in DFU care, where individual diagnostics and modifiable risk factors guide the right prevention support at the right time. Our findings extend this argument by grounding it in consumer experience: personalisation is not only a clinical aspiration but reflects what consumers identify as missing from current prevention. Personalisation at this level can only happen when consumers are partners in shaping their own prevention strategies. The implication for prevention research is that interventions should be developed and tested in ways that account for individual variation, including differences in clinical history, life circumstances, and self‐management capability.

The top voted strategies were dominated by wearable sensors and AI, with structured education also ranking highly. This cuts against the assumption that older consumers, those from CALD backgrounds, and those from areas of socioeconomic disadvantage are technology resistant [18, 19]. In our study, these consumers actively prioritised technology as the mechanism for making prevention sustainable. That said, technology‐enabled prevention must be designed for the population's needs and access, including low‐cost, culturally safe, and community‐delivered options. Current guidelines place the work of prevention on the individual: examining the feet daily, recognising early skin breakdown such as cuts, heel fissures, and cracks, and maintaining a daily emollient routine [1]. Consumers prioritised technology to offload this burden. Wearable sensors externalise the work of detecting subtle changes [15], while AI powered learning adapts to the person rather than requiring the person to adapt to generic content [20]. Each strategy targets the same underlying problem: prevention currently relies on sustained vigilance, accurate self‐interpretation, and uninterrupted motivation, all of which are difficult to maintain alongside work, fatigue, and competing health demands [21]. Technology enabled strategies should therefore not be treated as add ons to existing prevention models [17]. They are the mechanism through which prevention can be delivered at scale, and through which personalisation, including individualised risk signals, learning, and feedback, becomes possible.

Consumers should be partners in setting their own personalised DFU prevention strategies, because they see prevention differently from clinicians and national bodies. Consumers prioritised what they wanted from prevention differently, focussing on the daily, personal work of prevention rather than the structural goals (access to care, provision of safe quality care, and research and development) that anchor the 2030 Australian Strategy [13]. They also defined prevention success differently, not by clinical endpoints but by living well, maintaining function, confidence, and quality of life [22]. The implication is that personalised prevention strategies are more likely to work when they are developed with the consumer rather than for them. In practice, this means setting prevention targets with the consumer, agreeing how those targets will be tracked, adjusting strategies when they are not met, and ensuring the targets reflect the consumer's own definitions of success, an approach consistent with shared decision making in other chronic conditions, where it has been linked to better adherence and outcomes [19, 20, 21, 22]. This is particularly important for socioeconomically disadvantaged and CALD communities, where the daily realities of prevention are hardest and the gap between standard prevention guidance and lived experience is widest [10].

This study has several limitations. Rapid qualitative analysis was used to allow emerging findings to inform subsequent workshops, which may have limited the depth of interpretation compared to more detailed analytic approaches such as in vivo coding or line by line thematic analysis [15]. This trade‐off was deliberate and necessary for the iterative co‐design structure, and rigour was supported through researcher and data triangulation across transcripts, field notes, and structured workshop outputs [16]. The small group consensus process may have been influenced by dominant voices, particularly where cultural and power dynamics are present. This was mitigated through experienced facilitation by a researcher (KC) not involved in participants' clinical care, individual ranking tasks alongside group discussion, and independent field notes recorded by two researchers (JNM, AMK). Finally, all workshops were conducted at a single hospital site in South East Queensland, which may limit transferability.

## 5 Conclusion

This co‐design study generated a consumer‐derived prevention research agenda with consumers at high risk of DFU, including Aboriginal and Pacific Islander participants and those from areas of socioeconomic disadvantage. Consumers consistently prioritised technology enabled implementation strategies across all three priority research directions. They also made clear that these strategies will only work if personalised to individual circumstances, and defined prevention success as living well, maintaining function, confidence, and quality of life. Together, these findings show that effective DFU prevention must be technology enabled, personalised, and developed in partnership with the consumer. Future prevention research should develop and evaluate strategies that meet these conditions, particularly with and for the communities carrying the greatest prevention burden.

## Author Contributions

J.N.M. and J.W. conceived the study. J.N.M. and K.C. designed the study. D.M.G.W., M.N., and M.A.K. contributed to participant recruitment. K.C. facilitated all workshops. A.M.K. took live notes during the workshops. J.N.M., A.M.K., and K.C. conducted the qualitative analysis. J.N.M. drafted the manuscript. All authors contributed to manuscript revision, read, and approved the final version.

## Conflicts of Interest

The authors declare no conflicts of interest.

## Data Availability

The data that support the findings of this study are available from the corresponding author upon reasonable request.
